# CD47-mediated regulation of glucose and lipid metabolism: implications for the pathogenesis of MASLD

**DOI:** 10.3389/fendo.2025.1535382

**Published:** 2025-06-24

**Authors:** Xinru Jiang, Wei Zhao, Botao Shen, Yumeng Han, Kexin Chen

**Affiliations:** ^1^ Department of Cardiology, The First Hospital of Jilin University, Changchun, China; ^2^ Core Facility of the First Hospital of Jilin University, Changchun, China

**Keywords:** CD47, MASLD, glucose metabolism, lipid metabolism, mitochondrial, hepatic sinusoidal, senescence

## Abstract

Metabolic dysfunction-associated steatotic liver disease (MASLD), previously known as non-alcoholic fatty liver disease (NAFLD), has gradually become a leading cause of end-stage liver disease as a heterogeneous group of diseases. While the underlying mechanisms of MASLD remain incompletely understood, it is clear that glycolipid metabolism, coupled with subsequent disruptions in hepatic sinusoidal homeostasis and cellular senescence play significant roles in its onset and progression. In recent years, CD47 has been recognized not only as a critical target in cancer therapy but also as a participant in the development of metabolic diseases through complex signaling pathways. Increasing evidence suggests that CD47 is closely associated with the development of MASLD; however, its role in MASLD has not yet been widely explored. Therefore, this review aims to summarize current research on the potential role of CD47 in the pathogenesis of MASLD, particularly in relation to disturbances in glucose and lipid metabolism.

## 1 Introduction

In recent years, with the increasing prevalence of obesity and insulin resistance (IR) in the population, the incidence of metabolic dysfunction-associated steatotic liver disease (MASLD) has been rising. Recent data reveal that the global prevalence of MASLD has grown from 25.3% between 1990 and 2006 to 38.0% during the period 2016–2019. At present, MASLD is considered the most prevalent chronic liver disease worldwide (1). MASLD was previously known as non-alcoholic fatty liver disease (NAFLD). In 2023, three leading liver associations proposed replacing the NAFLD with MASLD, and renaming non-alcoholic steatohepatitis (NASH) to metabolic dysfunction-associated steatohepatitis (MASH), highlighting the importance of metabolic dysfunction (2). Despite the differences in definitions between MASLD and NAFLD, it is widely believed that data from NAFLD studies are also applicable to MASLD (3).

MASLD is defined by the accumulation of lipid droplets in more than 5% of hepatocytes, along with at least one cardiometabolic risk factor, such as obesity, diabetes, dyslipidemia, or hypertension, while ruling other causes of fatty liver disease. Additionally, affected individuals are generally non-drinkers, with alcohol intake below 20 g/day for women and 30 g/day for men (4). MASLD encompasses a range of liver disease states, ranging from simple hepatic steatosis to more severe forms involving hepatic inflammation and hepatocyte ballooning, referred to as metabolic dysfunction-associated steatohepatitis (MASH). It can progress to varying degrees of fibrosis, potentially culminating in cirrhosis and significantly increasing potentially culminating in cirrhosis and significantly increasing the risk of hepatocellular carcinoma (5).

CD47 is a transmembrane protein of the immunoglobulin superfamily that is almost universally expressed on the surface of human cells. It has a crucial role in regulating cellular processes such as renewal, adhesion, apoptosis, and phagocytosis. With a molecular weight of approximately 50 kDa, CD47 belongs to the immunoglobulin superfamily and exists in four isoforms. Its molecular structure includes an N-terminal extracellular immunoglobulin variable (IgV) domain, five transmembrane domains, and a C-terminal cytoplasmic tail that undergoes alternative splicing (6). In 1987, this protein was initially identified as a complex of Rh antigens on red blood cells and was later found to copurify with integrin αVβ3. Molecular cloning subsequently revealed that the protein is identical to the cancer antigen OV-3, leading to its alternative names, integrin-associated protein (IAP) and ovarian tumor marker (OA3) (7). In 2000, CD47 was identified as a marker that enables red blood cells to evade phagocytosis by binding to the signal regulatory protein alpha (SIRPα) on phagocytes. This interaction transmits a “don’t eat me” signal, preventing the clearance of red blood cells by the immune system (8). This role has been extensively studied in the context of cancer, However, in recent years, increasing research has shown that CD47 is closely associated with metabolic diseases such as diabetes, coronary atherosclerosis, and MASLD (9, 10). Given the critical role of metabolic dysfunction in MASLD pathogenesis, we conducted this review to offer an in-depth analysis and synthesis of the available literature. The following sections will review the role of CD47 in MASLD, with particular emphasis on the mechanisms underlying disturbances in glucose and lipid metabolism.

## 2 The roles of CD47

CD47 is a ubiquitously expressed integral membrane protein that functions as a high-affinity receptor for secreted matricellular protein thrombospondin-1 (TSP1), while serve as a ligand for the inhibitory phagocyte receptor signal-regulatory protein-α (SIRPα). In addition, other molecules such as SIRPγ and integrins have also been implicated in CD47 interactions. Although SIRPγ binds CD47 with lower affinity and lacks classical signaling motifs, it can promote T cell adhesion and activation (11–13). CD47 also interacts in cis with integrins such as β1 and β3, influencing cell adhesion and migration through indirect regulation rather than classical ligand-receptor signaling (6). Therefore, the dual roles of CD47 exerts various cellular processes such as proliferation, angiogenesis, migration, apoptosis, differentiation, stress responses, and metabolism.

### 2.1 Signal inhibitory receptor protein- alpha

Signal regulatory protein alpha (SIRPα), the receptor for CD47, shares structural similarities with CD47. It consists of three extracellular immunoglobulin-like domains, a transmembrane domain, and a C-terminal intracellular domain, which includes two immunoreceptor tyrosine-based inhibitory motifs (ITIMs) (14). The single IgV domain of CD47 binds to the N-terminal domain of SIRPα, leading to tyrosine phosphorylation of the ITIMs on SIRPα. This phosphorylation then recruits and activates protein tyrosine phosphatases, inhibiting macrophage phagocytic activity. As a result, a “don’t eat me” signal is transmitted, preventing the cell from being engulfed and eliminated by phagocytes (15).

### 2.2 Thrombospondin-1

Thrombospondin-1 (TSP1) is an endogenous ligand of CD47 and a homotrimeric glycoprotein with multiple functional domains (16). With a molecular weight of approximately 420–450 kDa, each subunit of TSP1 includes an N-terminal heparin-binding domain, a central repeat region, and a C-terminal (COOH) domain that promotes cell adhesion (17). TSP1 is a major component of the α-granules released by platelets upon activation (18). In addition to its presence in platelets, TSP1 is expressed at low levels in nearly all human tissues, including the liver (19). Due to its multiple specialized domains, TSP1 can bind to various cell receptors and perform diverse functions (19). The interaction between the C-terminal domain of TSP1 and CD47 has a critical role in numerous pathways, including cell renewal, inflammatory responses, redox regulation, adipocyte function, and vascular endothelial function (16). This interaction is essential in both physiological and pathological contexts.

## 3 CD47 in MASLD

The primary characteristic of MASLD is the accumulation of triglycerides (TG) in the liver. While the mechanisms underlying the onset and progression of MASLD are not fully understood, the most widely accepted model to explain the development of MASLD and the progression from simple steatosis to MASH is the “two-hit” hypothesis, which suggests that the first hit is an imbalance in fatty acid metabolism, eventually causing hepatic TG accumulation. The “second hit” leads to inflammation, hepatocyte injury, and fibrosis. Factors initiating the second hit are proinflammatory cytokines, oxidative stress and subsequent lipid peroxidation, adipokines, and mitochondrial dysfunction (20). However, subsequent research has revealed that the pathogenesis of MASLD is far more complex than the two-hit hypothesis suggests. Studies have found that factors, including oxidative stress, immunometabolism, disorders of glucose and lipid metabolism, intrahepatic cellular interactions, alterations in gut microbiota, and genetic susceptibility collectively contribute to the development and progression ofMASLD/MASH (21). Accumulating evidence indicates that metabolic disorders, including obesity, diabetes, insulin resistance, dyslipidemia, metabolic syndrome, and hyperuricemia, play critical roles in the development and progression of MASLD/MASH. Disturbances in glucose and lipid metabolism affect mitochondrial function, hepatic sinusoidal homeostasis, oxidative stress, and cellular senescence, with CD47 playing a crucial role in these processes. This review will primarily focus on glucose and lipid metabolism and provide an overview of the role of CD47 in the associated mechanisms. **Table 1** summarizes selected representative studies.

**Table 1:** Preclinical evidence of CD47 function in MASLD: insights from animal and organoid models.

Animal Model	Intervention	Major Findings
CD47-deficient mice (C57BL/6, male, 8 weeks); fed high-fat diet (60% kcal fat) or low-fat diet (10%) (22).	Global CD47 knockout (Jackson Laboratory)	1.CD47 deficiency promotes brown adipocyte differentiation via cGMP/PKG signaling, enhancing energy expenditure and reducing diet-induced obesity. 2.CD47-deficient mice exhibit reduced lipid droplet accumulation in brown adipose tissue, increased expression of UCP1 and CPT1b, decreased macrophage infiltration in adipose tissue, attenuated inflammation, reduced hepatic steatosis, and improved glucose tolerance and insulin sensitivity.
CD47-deficient mice (C57BL/6J, male, 19 months); normal diet (23).	Global CD47 knockout (Jackson Laboratory)	1. CD47-deficient mice exhibit increased UCP1 expression in white adipose tissue. 2. Brown adipose tissue in CD47-deficient mice retains a youthful morphology, with upregulation of mitochondrial and metabolism-related genes. 3. Following cold exposure, CD47-deficient mice show increased expression of UCP1, Dio2, and PGC1α in subcutaneous white adipose tissue.
Diet-induced obesity (DIO) mice: C57BL/6, male, 6 weeks; HFD for 6 weeks ob/ob mice: C57BL/6J-Lepob/Lepob, male, 6 weeks (24).	CD47 antisense oligonucleotide (ASO)	1. CD47 ASO reduces weight gain, fat mass, improves glucose tolerance, and alleviates steatosis. 2. CD47 ASO treatment did not significantly alter the expression of UCP1, CPT1, or PGC1α in brown adipose tissue; cold tolerance remained unchanged, and no browning of white adipose tissue was observed. 3. Reduced inflammatory cytokines (TNF-α, IL-1β), enhanced M2 macrophage polarization, CD47 ASO treatment also decreased CD8 expression in epididymal white adipose tissue.
AMLN-induced NASH model: C57BL/6, male; 20 weeks of diet (40% fat, 20% fructose, 2% cholesterol) 3D human NASH organoid model: hepatocytes + THP-1-derived macrophages + hepatic stellate cells, exposed to palmitate, high glucose, LPS (13).	Anti-CD47 antibody	1. Reduced hepatic inflammation and immune cell infiltration; lowered proinflammatory cytokines. 2. Decreased expression of fibrotic markers (α-SMA, collagen I). 3. Human model confirms anti-CD47 efficacy in suppressing inflammation and fibrosis.
CD47 KO vs. WT C57BL/6, male, 8 weeks; fed either low-fat (10%) or high-fat diet (45%) for 40 weeks (25).	Global CD47 knockout (Jackson Laboratory)	CD47 deficiency enhances NF-κB activation, elevates IL-1β, TNFα, IL-6, reduces IL-10, increases CCL2 and macrophage infiltration, leading to exacerbated steatosis, inflammation, and fibrosis.
1.FPC-induced NASH (fructose-palmitate-cholesterol diet, 16 weeks) 2.HF-CDAA-induced NASH (high-fat choline-deficient L-amino acid diet, 12 weeks) 3.AP20187-induced hepatocyte necroptosis model (26).	Anti-CD47 antibody, anti-SIRPα antibody, or AAV8-H1-shCD47	Blocking CD47 or SIRPα promotes macrophage clearance of necHCs and reduces HSC activation (α-SMA, Col1a1); anti-SIRPα avoids anemia associated with CD47 blockade.
HFD-induced liver fibrosis model: C57BL/6J, male; normal vs. 60% fat diet, 20 weeks (27).	CD47 silencing in HSCs (siRNA)	1.YAP/TEAD4 binds CD47 promoter, drives its transcription. Inhibition of CD47 or YAP/TEAD4 reduces fibrotic markers. 2.CD47 activates AKT/mTOR, inhibits HSC apoptosis, enhances proliferation, promoting fibrosis.
Male Wistar rats for isolation of LSECs (28).	CD47 blocking antibody	TSP1 activates Rho/ROCK via CD47, leading to myosin phosphorylation, LSEC fenestrae contraction, and defenestration.
Brown adipose tissue-specific CD47 knockout mice, both male and female mice were included for sex-based comparisons. CD47^fl/fl^ mice on a C57BL/6 background served as controls (29).	CD47 conditional deletion in brown adipocytes	Metabolic benefits observed in males: reduced weight, hepatic steatosis, improved glucose control without altered thermogenesis. No significant effects in females, suggesting sex-dependent CD47 function in BAT.

This table summarizes representative studies and does not encompass all available literature on the subject. BAT, brown adipose tissue; WAT, white adipose tissue; sWAT, subcutaneous white adipose tissue; eWAT, epididymal white adipose tissue; UCP1, uncoupling protein 1; CPT1b, carnitine palmitoyltransferase 1B; cGMP, cyclic guanosine monophosphate; PKG, cGMP-dependent protein kinase; DIO, diet-induced obesity; HFD, high-fat diet; LFD, low-fat diet; IL-1β, interleukin-1 beta; TNF-α, tumor necrosis factor alpha; IL-6, interleukin-6; IL-10, interleukin-10; CCL2, chemokine (C-C motif) ligand 2; AMLN, Amylin liver NASH diet (high-fat, high-fructose, high-cholesterol diet); α-SMA, alpha-smooth muscle actin; Col1a1, collagen type I alpha 1 chain; NF-κB, nuclear factor kappa-light-chain-enhancer of activated B cells; KO, knockout; WT, wild-type; NASH, nonalcoholic steatohepatitis; HSCs, hepatic stellate cells; LSECs, liver sinusoidal endothelial cells; TSP1, thrombospondin-1; ROCK, Rho-associated protein kinase; AKT, protein kinase B; mTOR, mammalian target of rapamycin; YAP, yes-associated protein; TEAD4, TEA domain family member 4; ASO, antisense oligonucleotide; AAV8, adeno-associated virus serotype 8; siRNA, small interfering RNA.

### 3.1 Disorder of glycolipid metabolism

**Figure 1:**
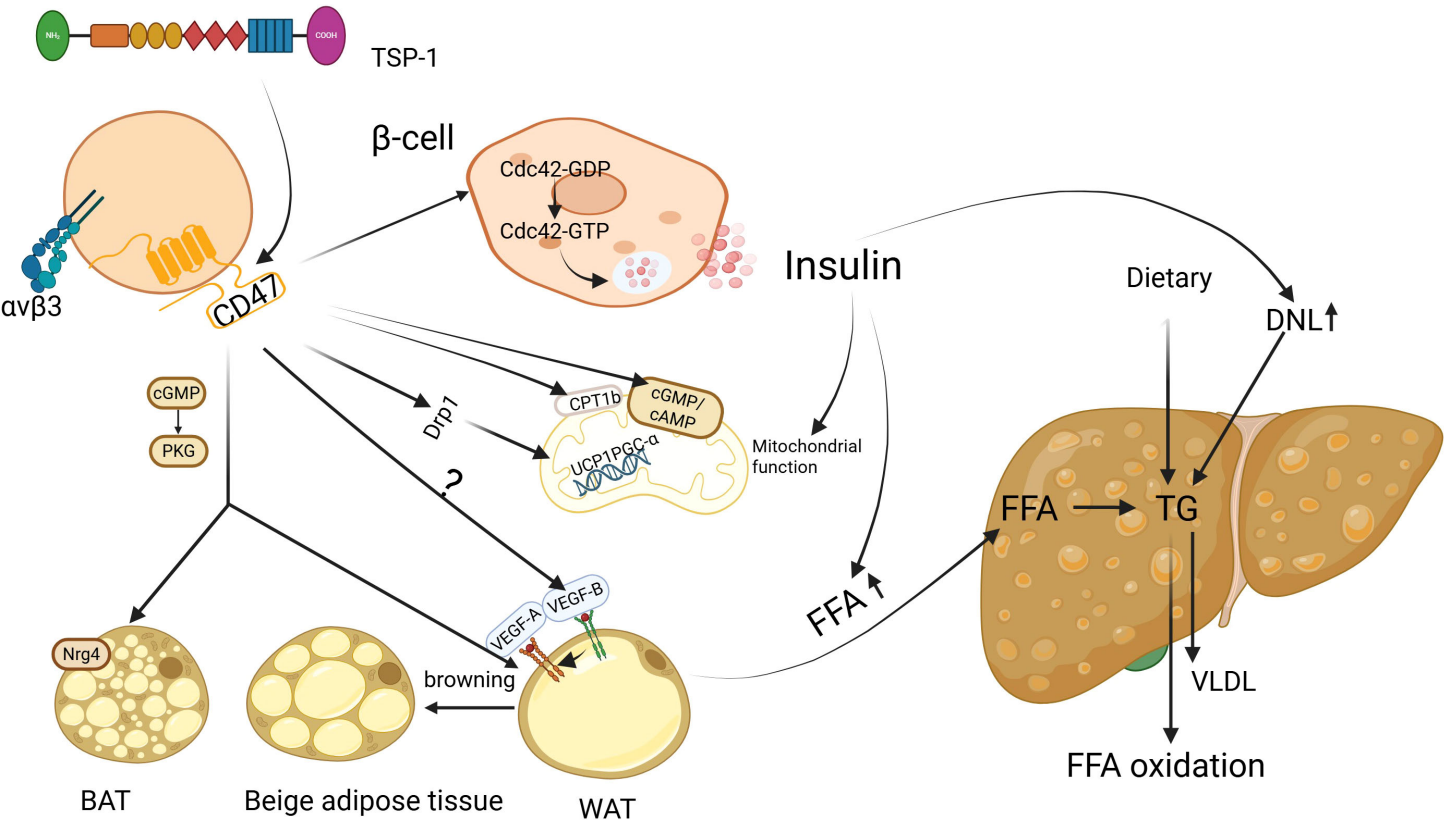
CD47 regulation of glucose and lipid metabolism in MASLD. Insulin resistance (IR) is recognized as a critical factor in the development of metabolic dysfunction-associated steatotic liver disease (MASLD). In the presence of IR, fat accumulation and mitochondrial dysfunction are observed. Suppressing CD47 expression has the potential to improve both lipid and mitochondrial function via multiple mechanisms, thereby reducing hepatic lipid accumulation. Additionally, this inhibition may help mitigate insulin resistance and the cascade of metabolic disturbances associated with it. BAT, brown adipose tissue; WAT, white adipose tissue; FFA, free fatty acids; TG, triglycerides; DNL, *de novo* lipogenesis; VLDL, very-low-density lipoprotein; Cdc42, cell division cycle 42; GDP, guanosine diphosphate; GTP, guanosine triphosphate; cGMP, cyclic guanosine monophosphate; AMP, cyclic adenosine mono-phosphate; PKG, protein kinase G; VEGF-A, vascular endothelial growth factor-A; VEGF-B, vascular endothelial growth factor-B; CPT1b, carnitine palmitoyl l transferase 1B; UCP1, Uncoupling protein 1;PGC-α, PGC-1 alpha; Drp1, Dynamin-related protein 1; Nrg4, neuregulin 4; Image created using the www.biorender.com.

MASLD is closely associated with metabolic dysfunction and is considered a manifestation of insulin resistance in the liver. Up to 95% of obese patients and 75% of diabetic patients may suffer from MASLD (30). The key feature of MASLD is the accumulation of triglycerides (TG) in the liver. The sources of TG include dietary TG, TG synthesized from free fatty acids (FFA) through *de novo* lipogenesis (DNL) in the liver, and TG formed from FFA released by adipose tissue breakdown and transported to the liver. TG can be transported out of the liver as very low-density lipoprotein (VLDL) or metabolized via β-oxidation. In the context of insulin resistance, increased breakdown of white adipose tissue (WAT) leads to substantial release of FFA, which activates the DNL pathway. At the same time, impaired β-oxidation, decreased adiponectin secretion, and leptin resistance contribute to excessive accumulation of TG in the liver (31, 32). TSP1 has been shown to play an important role in IR (33), and CD47, as its ligand, has gained increasing attention for its involvement in IR (**Figure 1**). CD47 deficiency protects mice from glucose intolerance and insulin resistance by diet and aging (22, 23). CD47 antisense oligonucleotide (ASO) treatment in two obesity mouse models (diet-induced obesity or genetically obese models) improved glucose homeostasis and hepatic steatosis (24). Moreover, recent research (34) has revealed that CD47 can inhibit insulin secretion through a new mechanism, by suppressing the activation of cell division cycle 42(Cdc42), a small GTPase in the Rho family involved in actin remodeling and vesicle exocytosis. Inhibiting CD47 expression enhances β-cell function by increasing pancreatic islet size and β-cell proliferation through the upregulation of c-myc expression, thereby maintaining glucose homeostasis and insulin sensitivity. These findings highlight the multifaceted role of CD47 in modulating insulin secretion, glucose homeostasis, and hepatic lipid metabolism.

The human body primarily contains three types of adipose tissue: WAT, brown adipose tissue (BAT), and beige adipose tissue. WAT, particularly visceral adipose tissue, functions as an endocrine organ, influencing liver metabolism by secretion of regulatory factors such as adiponectin, leptin, and interleukin -6(IL-6), On the other hand, BAT and beige adipose tissue generate heat through uncoupled oxidative phosphorylation. Increased BAT activity can potentially improve metabolic disturbances and reduce hepatic steatosis (35). Under certain conditions, WAT can convert into beige adipose tissue, a process known as browning. Thus, the balance between WAT and BAT function plays a crucial role in regulating liver lipid homeostasis and MASLD progression.

Under conditions of IR, the levels of FFA increase, leading to heightened energy expenditure by brown adipocytes in an attempt to lipid metabolic balance. However, when FFA levels continue to rise and the oxidative capacity of brown fat cells fails to match it, excessive lipid accumulation occurs (36). Also, adipose tissue dysfunction is considered a key mechanism in developing MASLD. For example, studies have shown that patients with MASLD exhibit reduced BAT activity, and the degree of hepatic lipid accumulation is influenced by BAT activity (35). This is thought to be primarily related to the thermogenic capacity of BAT and its secretion of regulatory factors, such as neuregulin 4 (Nrg4), which exerts its effects by activating epidermal growth factor receptor 3 (ErbB3) and epidermal growth factor receptor (ErbB4) signaling pathways in hepatocytes, helping counteract diet-induced hepatic steatosis (37). These observations suggest that targeting CD47 may improve systemic metabolism by enhancing BAT function and WAT browning.

During adipocyte browning, mitochondrial biogenesis increases alongside upregulation of uncoupling protein 1 (UCP1). Cold exposure or norepinephrine can induce PGC-1 alpha (PGC-1α), expression via activation of the β3-adrenergic receptor and cAMP/PKA pathway. Similarly, AMPK activation in response to energy stress, physical exercise, or pharmacological agents also promotes this process (38–40). CD47 is significantly upregulated in the brown adipose tissue of mice with high-fat diet-induced obesity. CD47 deficiency enhances energy expenditure by promoting brown adipocyte differentiation through upregulation of the cGMP/PKG signaling pathway, thereby alleviating obesity induced by a high-fat diet in these mice (22). In aged CD47-deficient mice, although there was no significant change in fat mass compared to wild-type mice, smaller epididymal white adipocytes were observed. In addition, there was an enhancement in the expression of (UCP1, type 2 deiodinase (Dio2), and PGC-1α, indicating enhanced browning of WAT. This adjustment protected the mice from obesity and glucose intolerance associated with aging (23). Notably, these effects displayed sexual dimorphism, with male mice showing more pronounced improvements in body weight regulation and lipid metabolism compared to females (29). Thus, CD47 deficiency appears to improve systemic metabolism by promoting BAT activation and WAT browning, offering protection against diet- and age-related metabolic disorders. Functionally, CD47 signaling intersects with β-adrenergic and AMPK pathways, converging on PGC-1α as a common downstream target. These findings suggest that CD47 may act as a regulatory node within classical browning networks, contributing to adipose tissue remodeling and the maintenance of energy balance.

Additionally, a recent study demonstrated that inhibiting vascular endothelial growth factor-B (VEGF-B) signaling can prevent the development of MASLD by blocking lipolysis in WAT (41). VEGF-B, a member of the VEGF family, is not only involved in angiogenesis but is also thought to be a crucial regulator of lipid metabolic disorders and glucose dysregulation (42–44). The expression level of VEGF-B is significantly elevated in patients with MASLD compared to those without the disease (45). VEGF-B exerts a lipid-lowering effect by binding to vascular endothelial growth factor receptor 1 (VEGFR1), activating adenosine monophosphate-activated protein kinase (AMPK), and partially through the indirect activation of the VEGF-A/VEGFR2 pathway (46, 47). It is well established that the binding of TSP1 to CD47 inhibits VEGFR2 activation and its downstream signaling, thereby suppressing nitric oxide (NO) production and cyclic guanosine monophosphate (cGMP) signal transduction (48). Both CD47 and VEGF-B regulate lipid metabolism by modulating lipolysis in white adipose tissue, with a partial overlap in their signaling pathways. However, whether CD47 can influence lipid metabolism via VEGF-B remains to be further explored. Also, a recent study (49) found that the Nrg4 expression level was remarkably elevated in brown fat of TSP1 knockout mice, The absence of Nrg4 has been shown to accelerate liver damage, fibrosis, inflammation, and cell death in a NASH mouse model. These findings suggest that the TSP1-CD47 axis may serve as a key regulator of adipose-liver crosstalk, influencing both lipid metabolism and hepatic steatosis in MASLD.

### 3.2 Mitochondrial dysfunction

Mitochondria are well-known as the primary sites of aerobic respiration and are fundamental to cellular energy metabolism and apoptosis. The liver, which contains an abundance of mitochondria, relies on these organelles to metabolize energy efficiently. When the body ingests excess lipids, the enhanced capacity of the mitochondrial tricarboxylic acid (TCA) cycle and β-oxidation help prevent the progression from obesity to MASLD (50). However, as hepatic steatosis progresses, the persistent excess of free fatty acids (FFAs) leads to mitochondrial dysfunction, characterized by impaired β-oxidation, reduced respiratory chain activity, alterations in mitochondrial morphology and membrane permeability, and compromised mitophagy (51, 52). These changes result in increased reactive oxygen species (ROS) production, contributing to lipid accumulation, inflammation, necrosis, and fibrosis in the liver, which in turn leads to a vicious loop of MASLD and mitochondrial dysfunction. Current evidence suggests that the signaling strength of the TSP1-CD47 pathway is inversely correlated with mitochondrial quantity and function within cells (53). The TSP1-CD47 pathway downregulates cGMP and cyclic adenosine monophosphate (cAMP), affecting mitochondrial biogenesis. Frazier et al. found that the expression levels of mitochondrial-related genes, such as PGC-1α, cytochrome b and c(cytb/c), and nuclear respiratory factor 1(NRF-1), are significantly elevated in the skeletal muscle of the CD47-null C57Bl/6J mouse model (54). This suggests that CD47 plays a crucial role in regulating mitochondrial function, and its absence may promote mitochondrial biogenesis, potentially offering therapeutic avenues for MASLD.

CD47 may also lead to the loss of mitochondrial membrane potential, increased ROS production, and promotion of cell death (55–57). Dynamin-related protein 1 (Drp1) has been shown to regulate mitochondrial-dependent cell death signaling pathways, and the binding of CD47 with TSP1 can promote Drp1 translocation from the cytoplasm to the mitochondria (13, 58). Another study (22) found an increased rate of mitochondrial uncoupling by upregulating the mRNA levels of UCP1 and carnitine palmitoyltransferase 1B (CPT1b) in BAT of CD47-null mice compared to wild-type mice. This enhanced mitochondrial uncoupling improved diet-induced hepatic steatosis. In summary, CD47 regulates mitochondrial dynamics and function, and its deficiency appears to have protective effects on mitochondrial activity, potentially mitigating liver steatosis.

### 3.3 Hepatic sinusoidal homeostasis

**Figure 2:**
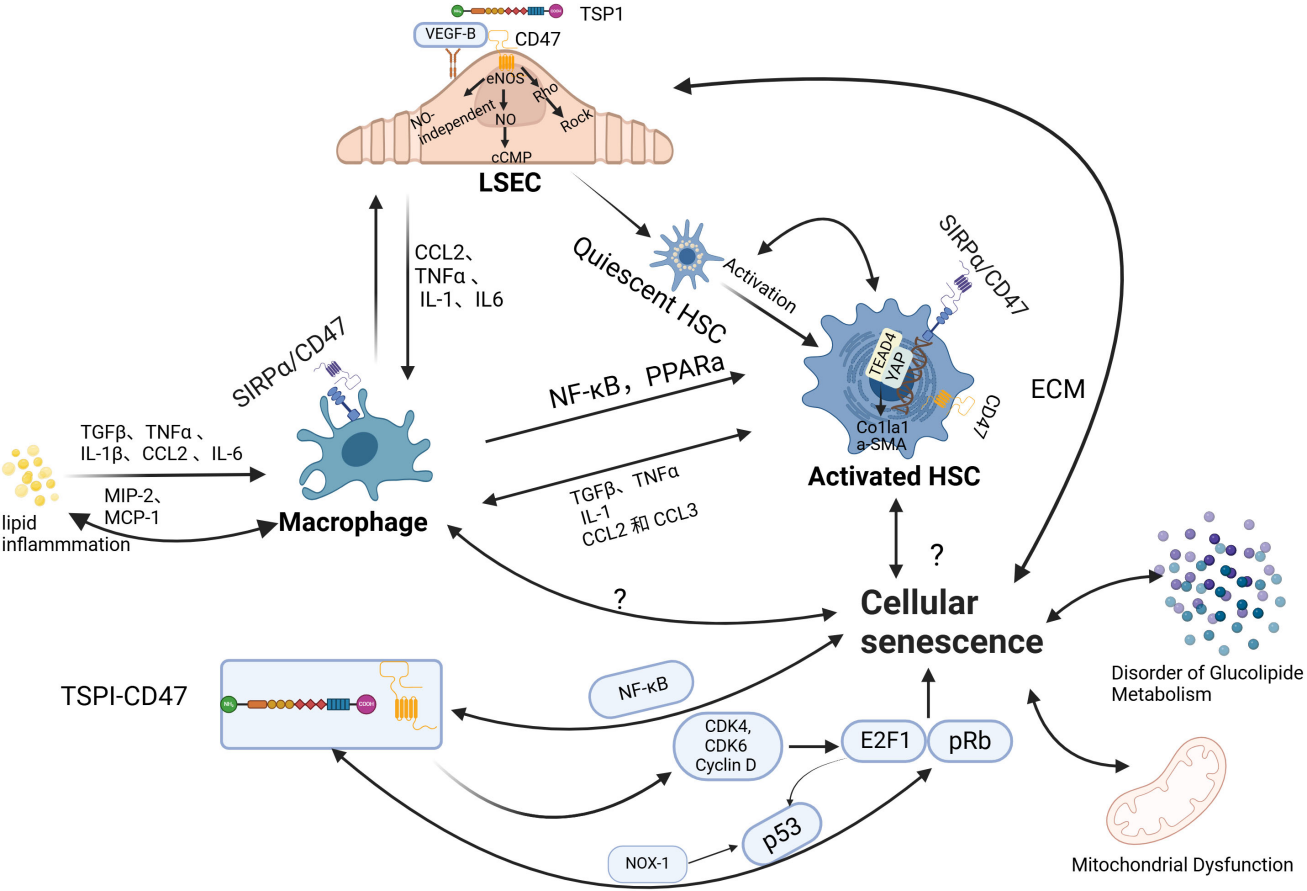
The role of CD47 in regulating hepatic sinusoidal function and cellular senescence. (1) liver sinusoidal endothelial cells (LSECs) play a pivotal role in maintaining sinusoidal homeostasis, supporting the normal function of hepatic stellate cells (HSCs) and macrophages. Under physiological conditions, LSECs exhibit anti-inflammatory and anti-fibrotic properties. However, phenotypic alterations in LSECs can lead to HSC activation and exacerbate macrophage infiltration. The maintenance of LSEC phenotype stability is dependent on nitric oxide (NO). The binding of TSP1 to CD47 inhibits vascular endothelial growth factor receptor 2 (VEGFR2) activation and its downstream signaling, reducing NO production. This, in turn, induces LSEC defenestration through myosin activation via the Rho-ROCK pathway. Additionally, studies suggest that targeting CD47 can modulate macrophage and HSC function via pathways such as Signal regulatory protein alpha (SIRPα) and YAP/TEAD4, thereby reducing hepatic inflammatory infiltration and fibrosis. (2) Aging contributes to the progression of metabolic dysfunction-associated steatotic liver disease (MASLD)/metabolic dysfunction-associated steatohepatitis (MASH) through various mechanisms, including its effects on hepatic glycolipid metabolism, mitochondrial function, and intrahepatic cellular processes. The interaction between TSP1 and CD47 influences the pRb-E2F1 and p53 pathways, thereby regulating the cell cycle. Nuclear factor-κB (NF-κB), a central regulator of inflammatory responses, is also modulated by CD47, with CD47 levels positively correlated with NF-κB expression. LSEC, liver sinusoidal endothelial cell;TSP1, thrombospondin-1; eNOS, endothelial nitric oxide synthase;NO, nitric oxide;cGMP, cyclic guanosine monophosphate;Rho, Ras homolog (GTPase);Rock, Rho-associated, coiled-coil-containing protein kinase;CCL2, C-C motif chemokine ligand 2;TNFα, tumor necrosis factor alpha;IL-1, interleukin-1;IL6, interleukin-6;NF-κB, nuclear factor-κB;PPARα, peroxisome proliferator-activated receptor alpha;HSC, hepatic stellate cell;TGFβ, transforming growth factor beta;MIP-2, macrophage inflammatory protein 2;MCP-1, monocyte chemoattractant protein 1;CCL3, C-C motif chemokine ligand 3;YAP1, Yes-associated protein 1;TEAD1, TEA domain transcription factor 1;Col1a1, collagen I chain;α-SMA, alpha-smooth muscle actin;SIRPα, signal regulatory protein alpha; ECM, extracellular matrix; CDK4, cyclin-dependent kinase 4; CDK6, cyclin-dependent kinase 6; E2F1, E2F transcription factor 1; pRb, phosphorylated Rb; NOX-1, NADPH oxidase 1; p53, tumor protein p53; Image created using the www.biorender.com.

Liver sinusoidal endothelial cells (LSECs), hepatic stellate cells (HSCs), and hepatocyte macrophages, primarily Kupffer cells (KCs), are key components of the liver sinusoids. These cells work in concert to maintain the integrity and function of the unique hepatic microcirculatory system. In conditions of IR or lipid metabolism disorders, the accumulation of lipids and their metabolites in the liver disrupts the hepatic sinusoidal microenvironment, leading to damage and dysfunction of liver sinusoidal endothelial cells. This disruption of hepatic sinusoidal homeostasis further promotes hepatic fat deposition, thereby creating a vicious cycle that plays critical roles in the pathogenesis of MASLD and MASH (**Figure 2**).

#### 3.3.1 Macrophage infiltration

Liver macrophages are primarily composed of resident Kupffer cells and monocyte-derived macrophages, which can be classified into pro-inflammatory M1 and anti-inflammatory M2 phenotypes. Monocyte recruitment is mainly regulated by CC chemokine receptor 2 (CCR2), while Kupffer cells are regulated by C-C motif chemokine ligand 2 (CCL2). Studies have shown that monocyte-derived macrophages exhibit more pronounced pro-inflammatory properties compared to resident Kupffer cells (59). Inflammatory cytokines can activate macrophages, leading to the increased release of macrophage inflammatory protein 2 (MIP-2) and monocyte chemoattractant protein 1(MCP-1), which further exacerbate macrophage infiltration in the liver (60). This process mainly involves nuclear factor-κB (NF-κB) and peroxisome proliferator-activated receptor alpha (PPARα) (61, 62). Macrophages can be activated by fatty acids, excess cholesterol, and their metabolites (such as leptin and adiponectin), resulting in the release of tumor necrosis factor (TNF-α) and interleukin-1 (IL-1), which affect hepatocyte function and activity (63). In MASLD, a distinct subset of liver lipid-associated macrophages (LAMs) expressing TREM2 is predominantly localized within steatotic regions and closely correlates with disease severity. TREM2 plays a critical role in the clearance of apoptotic cells and lipid metabolism regulation, and its deficiency exacerbates hepatic inflammation. Bariatric surgery has been shown to improve MASH progression by enhancing the reparative functions of TREM2^+^ macrophages (64). In addition to macrophages, adaptive immune cells such as CD8^+^ T cells and Th1/Th17-polarized CD4^+^ T cells contribute to liver inflammation by secreting IFN-γ and TNF-α. B cells also promote disease progression via antibody production and pro-inflammatory cytokine release. Moreover, natural killer (NK) cells and neutrophils participate in hepatocyte injury and inflammatory responses (65–67).

CD47 deficiency has been associated with decreased levels of the pro-inflammatory cytokines TNF-α and interleukin-6(IL-6), and higher levels of the anti-inflammatory cytokine interleukin-10 (IL-10) in mice fed with a high-fat diet. This reduction in inflammation correlates with lower levels of MCP-1 and CCR2, leading to decreased macrophage infiltration in adipose tissue (22). Similar findings have been corroborated by *in vitro* studies (22) Moreover, anti-CD47 therapy has been demonstrated to inhibit MCP-1 expression and secretion, resulting in diminished liver infiltration of monocytes/macrophages. Additionally, this therapy reduces hepatic stellate cell activation via transforming growth factor-beta (TGF-β) signaling, thereby alleviating hepatic inflammation and fibrosis (13). A recent study (58) reported that the expression levels of CD47 on necrotic hepatic apoptotic cells (necHCs) and SIRPα on liver macrophages are elevated, impairing the macrophages’ ability to clear necHCs. This impairment contributes to the exacerbation of liver fibrosis and inflammatory infiltration. Blocking the CD47-SIRPα axis has been demonstrated to promote the phagocytic clearance of necHCs by liver macrophages, thus inhibiting the progression of liver fibrosis. Besides, Anti-CD47 therapy has been shown to reduce neutrophil infiltration in both circulation and liver tissue in NASH models, and to inhibit neutrophil migration (13). Additionally, CD47 blockade suppresses dendritic cell maturation and activation, modulates T cell responses, and promotes the differentiation of naïve T cells into regulatory T cells. In B cells, CD47 enhances maturation and activation, while CD47 expression in NK cells contributes to their recruitment and activation (68, 69). Overall, CD47 orchestrates the migration, activation, and apoptosis of various immune cell populations, working in concert with TREM2^+^ macrophages and other immune subsets to shape the hepatic inflammatory microenvironment and drive MASLD progression. These findings suggest that targeting CD47 has therapeutic potential for reducing liver inflammation and fibrosis by modulating immune responses and enhancing apoptotic cell clearance.

However, contradictory findings have emerged. In another study, CD47 knockout mice raised on a high-fat diet exhibited decreased expression of PPARα and sirtuin1(SIRT1)—a NAD+-dependent deacetylase that enhances PPARα activity, upregulates fatty acid oxidation (FAO), and downregulates lipogenic gene expression (70). These mice also showed increased phosphorylation, nuclear translocation of the NF-κB p65 subunit, and elevated hepatic CCL2 levels, leading to increased monocyte/macrophage infiltration (25). CD47 gene knockout and anti-CD47 antibody treatment exhibit distinct effects in liver disease models, likely due to differences in mechanisms of action and duration of intervention. Global and sustained CD47 deficiency resulting from genetic knockout may elicit compensatory responses that disrupt lipid metabolic homeostasis, potentially impairing lipid export via downregulation of apolipoproteins or undermining innate hepatic defense mechanisms against steatosis. In contrast, anti-CD47 antibody therapy primarily functions by blocking the CD47–SIRPα interaction, thereby enhancing macrophage-mediated clearance of apoptotic cells, reducing pro-inflammatory cytokine production, and inhibiting monocyte/macrophage infiltration and HSC activation. This form of therapy typically involves short-term and localized interventions, thereby avoiding long-term disturbances in lipid metabolism. This conflicting evidence underscores the complexity of CD47’s role in metabolic and inflammatory diseases, suggesting that CD47 may exert both beneficial and detrimental effects depending on the context. Further research is necessary to reconcile these findings and better understand the full spectrum of CD47’s involvement in liver pathology.

#### 3.3.2 Hepatic stellate cells

Hepatic stellate cells (HSCs) are non-parenchymal perisinusoidal cells that remain quiescent under normal conditions. However, upon stimulation by lipotoxic metabolites, inflammation, and oxidative stress, they become activated and transform into myofibroblasts. Activated HSCs secrete procollagen, a component of the extracellular matrix (ECM), pro-inflammatory, as well as pro-fibrotic cytokines, influencing surrounding cells via paracrine or autocrine signaling to promote liver fibrosis. It is currently believed that the persistent overactivation of HSCs is a key factor in liver fibrosis and a critical step in the progression to MASH (71). CD47 is significantly upregulated in activated HSCs. Previous research (26) by Shi et al. showed that blocking the CD47-SIRPα axis could alleviate diet-induced NASH-related liver fibrosis. Recently, Li et al. reported that CD47 knockdown reduced the expression of alpha-smooth muscle actin (α-SMA) and collagen I(COL1A1)by inhibiting the AKT/mTOR signaling pathway in a high-fat diet-induced mouse model of MASLD, thus preventing HSC activation and reducing liver fibrosis, similar to previous findings. The study further suggested that this process may be mediated by the YAP/TEAD4/CD47 signaling axis (27). Yes-associated protein (YAP), a key transcriptional regulator in the Hippo pathway, plays a pivotal role in liver regeneration and fibrogenesis. Notably, upregulation and nuclear translocation of YAP have been shown to activate HSCs both *in vitro* and *in vivo* (72). One of YAP’s canonical target genes, connective tissue growth factor (CTGF), is significantly overexpressed in fibrotic liver tissue and is capable of activating HSCs while promoting the synthesis and secretion of ECM proteins. CTGF also contributes to cell proliferation, migration, and phenotypic transformation, positioning the YAP–CTGF axis as a central driver of hepatic fibrogenesis (73, 74). Importantly, extensive crosstalk exists between the YAP/TAZ and TGF-β/Smad signaling pathways under various physiological and pathological conditions. Although direct interaction between YAP and Smad3 is relatively weak, YAP can form functional complexes through the transcriptional coactivator p300 and the transcription factor TEAD4, synergistically regulating the expression of pro-fibrotic genes such as CTGF and CYR61 (75–77). This interaction may represent a critical node in the integration of TGF-β and YAP/TEAD signaling.

Collectively, these findings indicate that the AKT/mTOR and YAP/TEAD4 pathways converge in mediating CD47’s role in HSC activation, and that their interplay with TGF-β/Smad signaling amplifies fibrogenic responses. but the interactions between these pathways remain unclear. Further research is needed to elucidate the specific regulatory mechanisms of CD47 in this process.

#### 3.3.3 Liver sinusoidal endothelial cells

LSECs are specialized endothelial cells that lack a basement membrane and possess small pores known as fenestrae. Under physiological conditions, LSECs are involved in lipid exchange between the blood and liver, keeping Kupffer cells and hepatic stellate cells inactive, modulating intrahepatic vascular resistance and portal vein pressure, all while exhibiting anti-inflammatory and anti-fibrotic properties (78). However, harmful stimuli such as FFA and microbial endotoxins can induce LSEC capillarization—a phenomenon characterized by the loss of fenestrae and the formation of a basement membrane—through the generation of reactive ROS and inflammation (79). This phenotypic change in LSECs impairs hepatic lipid uptake and metabolism, thereby promoting liver injury, inflammatory cell infiltration, and fibrosis (80).

The stability of the LSEC phenotype is closely associated with NO production. Also, LSECs can secrete NO to maintain the quiescence of hepatic stellate cells (81) VEGF is thought to be a crucial regulator of the LSEC phenotype, exerting its effects through both NO-dependent (eNOS-NO-cGMP) and NO-independent pathways (82). It is now clear that TSP1 binds to CD47 to inhibit VEGFR2 activation and downstream signaling, leading to reduced NO production and cGMP signal transduction (48). However, there is currently no direct evidence that blocking CD47 in NAFLD leads to increased NO secretion by LSECs, thereby improving fibrosis. Moreover, a study has demonstrated that the CD47-TSP1 pathway can induce LSEC defenestration through the activation of myosin by the Rho-ROCK pathway, and targeting CD47 has been shown to reduce LSEC defenestration (28). All these findings suggest that CD47 may influence the development and progression of MASLD by modulating both the function and morphology of LSECs.

### 3.4 Senescence

Aging is characterized by the gradual loss of physiological integrity and is a major risk factor for many diseases. Cellular senescence, considered one of the hallmarks of aging, is defined as a permanent cell cycle arrest state (83). While senescent cells lose their proliferative capacity, they gain an increased ability to secrete pro-inflammatory factors, a phenomenon known as the senescence-associated secretory phenotype (SASP), which affects surrounding cells through paracrine signaling and contributes to chronic inflammation (84). Although the causal relationship between MASLD and senescence remains unclear, it is undeniable that aging is closely linked to the occurrence and progression of MASLD/MASH. Recent research has gradually revealed that both aging and CD47 contribute to the development of MASLD/NASH by influencing glucolipid metabolic dysfunction, mitochondrial function, and hepatic sinusoidal homeostasis through several shared pathways (13, 22, 26).

Recently, a study (85) showed that aging WAT cells release increased levels of FFA, exacerbating hepatic steatosis. After treatment with senolytics (drugs that selectively eliminate senescent cells), the liver steatosis was alleviated by clearing the senescent adipocytes. Previous studies (86) have shown that CD47 deficiency can prevent aging-induced glucolipid metabolic dysfunction, thereby alleviating hepatic steatosis. The glucolipid metabolism disorders caused by senescent cells are currently thought to be related to mitochondrial metabolic dysregulation (87). As cells age, mitochondria may exhibit reduced energy production, weakened antioxidant capacity, and impaired autophagy, leading to abnormal lipid accumulation within cells (88, 89). In the aging liver, multiple cellular dysfunctions can be observed: 1) A reduction in the number of LSECs, with decreased expression of markers maintaining their normal morphology (VEGFR2, CD32b) and reduced vasodilation capacity (90). 2) Considering the heterogeneity of hepatic macrophages, the conclusions on the effect of aging on the phagocytic clearance capacity of hepatic macrophages are not uniform, and most studies now suggest that aging leads to an enhanced pro-inflammatory response in macrophages thereby leading to further deterioration of cellular function (91–93). 3) The specific role of senescent HSC on fibrosis is unclear; senescent HSC can ameliorate hepatic fibrosis, but a recent study showed that the number of senescent HSC increased in MASH and showed pro-fibrotic properties (94–96). Collectively, these findings highlight that aging-driven cellular senescence, particularly through dysregulated adipose tissue metabolism, mitochondrial dysfunction, and hepatic non-parenchymal cell impairment, plays a central role in the progression of hepatic steatosis and fibrosis.

Recent research has revealed that aging not only elevates the expression levels of TSP1 (54, 97–99) but also enhances the clustering of CD47 on the cell surface, thereby intensifying TSP1-CD47 signaling. Furthermore, several studies (9, 86, 99) have demonstrated that TSP1 can induce endothelial cell senescence via CD47. The phosphorylated Rb- E2F transcription factor 1 (pRb-E2F1) pathway is recognized as a crucial mechanism for cell cycle regulation, TSP1, acting as a downstream effector of E2F1, binds to CD47 on endothelial cells, inhibiting the activity of cyclin D1 and cyclin-dependent kinase 4/6(CDK4/6), activating the NADPH oxidase 1(Nox1) complex and playing an important role in the pRb pathway through p53-induced DNA damage response. In addition, E2F1 regulates the cell cycle by inducing p53 phosphorylation (99–102). Research (103, 104) has indicated that CD47 deficiency results in the reactivation of several stem cell markers, such as c-myc and SRY-Box transcription factor 2 (Sox2), in fully differentiated adult cells, enabling them to recapture some stem cell-like characteristics. As aging progresses, senescent cells accumulate in many tissues, but these cells are typically cleared by macrophages. When this clearance function is impaired, senescent cells accumulate, leading to a series of pathological processes. Macrophages play a critical role in reducing the number of senescent cells through phagocytosis (105). However, senescent cells increase CD47 expression, which binds to the SIRP-α molecule on macrophages, enhancing the “don’t eat me” signal. This not only inhibits macrophages from engulfing and phagocytosing these senescent cells, but also reduces their ability to clear surrounding cells (97). Additionally, NF-κB, known as the master regulator of inflammatory cytokines, has been identified as an activator of age-related transcriptional changes. Its expression is elevated in MASLD patients, and various stimuli can exacerbate liver steatosis via NF-kB signaling, Inhibition of its activity ameliorates hepatic steatosis (106–108). CD47 is positively correlated with NF-κB levels (109), and CD47 deficiency suppresses NF-κB activity, resulting in the downregulation of IL-1β, IL-6, and TNFα in the livers of aging mice (102). However, in CD47 knockout (CD47KO) mice, the phosphorylation and nuclear translocation of the p65 subunit of NF-κB were significantly enhanced, promoting the progression of MASLD and liver fibrosis (25). This mechanism parallels the upregulation of advanced glycation end-products (AGEs) with age, which accelerates triglyceride accumulation and, in turn, the development of MASLD (110). This seems to contradict previous research findings, CD47 regulates the development of MASLD/MASH through various pathways, and multiple stimuli can aggravate hepatic steatosis via the NF-kB signaling pathway. Experimental variables, including feeding duration and methods used to generate CD47-deficient mouse models, can impact these outcomes. In summary, accumulating evidence suggests that aging is an emerging risk factor for MASLD, and further research is needed to elucidate the specific mechanisms by which CD47 influences this process. Collectively, these findings suggest that CD47, by integrating signals from TSP1, NF-κB, and immune evasion pathways, plays a complex and context-dependent role in aging-related hepatic steatosis and fibrosis. Further clarification of these mechanisms will help resolve existing contradictions and guide targeted interventions.

## 4 Conclusions

The increasing prevalence of Metabolic dysfunction-associated steatotic liver disease (MASLD) will lead to a substantial global disease burden and public health costs, and its pathogenesis remains unclear. In this review, we summarize the regulatory role of CD47 in various pathogenic mechanisms of MASLD/MASH, with a focus on glucose and lipid metabolism. Although significant progress has been made in understanding the role of CD47 in metabolic dysfunction-associated steatotic liver disease (MASLD), key questions remain. The specific functions of CD47 in hepatocytes, liver sinusoidal endothelial cells, and hepatic stellate cells require further clarification, particularly regarding its context-dependent effects on inflammation and fibrosis. Its interactions with metabolic pathways—such as PI3K/AKT/mTOR, cGMP/PKG, NF-κB, and YAP/TEAD4—may uncover synergistic targets but remain insufficiently explored. Evidence also suggests a sex-dependent role of CD47 in lipid metabolism and aging-related disorders, highlighting the importance of sex-stratified research. To date, most findings linking CD47 to MASLD come from animal or *in vitro* studies, with limited validation in human tissues. While anti-CD47 antibodies show promise in oncology, their metabolic effects are unclear and raise safety concerns. Given CD47’s ubiquitous expression on erythrocytes, its blockade may disrupt “self” recognition and lead to hemolytic anemia—a major adverse event in clinical trials. Addressing these issues may help advance our understanding of the link between CD47 and MASLD and promote its clinical application in metabolic diseases.
